# Effect of Induction Heating Temperature on the Uniformity of Mechanical Properties of Bulb Flat Steel Sections in the Quenched State

**DOI:** 10.3390/ma18112626

**Published:** 2025-06-04

**Authors:** Zhen Qi, Xiaobing Luo, Fengrui Liang, Feng Chai, Qilu Ge, Zhide Zhan, Chunfang Wang, Wei Fan, Hong Yang, Yitong Liu

**Affiliations:** 1 Research Institute of Engineering Steels, Central Iron and Steel Research Institute Co., Ltd., Beijing 100081, China; qi152315zhen@163.com (Z.Q.);; 2 Experimental Research Center, Central Iron and Steel Research Institute Co., Ltd., Beijing 100081, China; 3 China State Shipbuilding Corporation Limited, Beijing 100081, China; 4 NCS Testing Technology Co., Ltd., Beijing 100081, China

**Keywords:** bulb flat steel, induction heating, uniformity of mechanical properties, strengthening mechanism

## Abstract

Induction quenching is critical for high-strength bulb flat steel, yet the influence of the heating temperature on mechanical property uniformity across sections remains underexplored. This study systematically investigates the effect of the induction heating temperature on mechanical property uniformity, prior austenite grain size, and microstructural evolution in bulb flat steel. Experimental results reveal that increasing the induction heating temperature from 845 °C to 1045 °C induces distinct mechanical responses: the yield strength disparity between the bulb and flat sections decreases by 93% (from 94 MPa), significantly improving sectional uniformity. Microstructural analysis indicates that prior austenite grain size coarsens with higher induction heating temperatures. The quenched microstructure comprises martensite and bainite in the bulb core, while the flat section is entirely martensitic. The yield strength differential between the bulb and flat sections is governed by temperature-dependent strengthening mechanisms: dislocation strengthening dominates at 845 °C~985 °C, with the bulb region exhibiting higher strength due to increased dislocation density, while grain boundary strengthening prevails at 1045 °C, where the flat region benefits from finer grains.

## 1. Introduction

In recent years, marine steel has progressively evolved towards higher strength and enhanced toughness [1,2,3,4,5]. Bulb flat steel is a crucial steel section that is welded to hull plates to create various structures aimed at increasing strength and deflection resistance, which has a significant impact on the safety and longevity of ship structures [6]. Research into and production of high-strength, high-toughness bulb flat steel can elevate the standards of marine equipment and foster the growth of the marine economy.

Bulb flat steel is an asymmetric section of steel consisting of a flat and a bulb [7]. Bulb flat steel has an irregular cross-sectional shape; the rolling process is an easily deformed non-uniformity, and the hot rolled state of the bulb flat steel strength is lower, so it is difficult to meet the requirements of a hot rolled state of high strength [6]. The non-uniform cross-sectional geometry of bulb flat steel renders it prone to warping and torsional deformation during conventional heating processes due to asymmetric thermal stress distribution. However, a quenching and tempering process (QT) is widely used to improve the comprehensive properties of alloy steel [8]. Induction heating offers significant advantages over conventional heating methods, including reduced processing time and minimized deformation. As a result, induction quenching has become a widely adopted technique in the heat treatment of high-strength spherical flat steel. The method ensures precise control over the hardening process, enhancing the material’s mechanical properties while maintaining dimensional stability. The efficiency and effectiveness of induction hardening make it particularly suitable for applications requiring high strength and durability in bulb flat steel components. Bulb flat steel is usually quenched by electromagnetic induction using a constrained method [9].

Induction heating is the use of electromagnetic induction and an eddy current in the workpiece heat generation process [10]. The utility model has the characteristics of high heating efficiency, large flexibility and easy precise temperature control. By customizing the shape and size of the induction coil, it is possible to effectively customize the heat treatment for parts with complex geometries [11]. Induction heating can increase the driving force of austenite nucleation and grain refinement advantages [12]. However, the eddy current effect makes the current and temperature generated inside the workpiece unevenly distributed, resulting in non-uniform properties of steel materials [13,14].

In terms of homogeneity of steel materials, many scholars have conducted relevant studies [15,16,17,18,19,20]. During the quenching process, the cooling rate is fast, and the residual austenite is enriched in the local area, which leads to the uneven distribution of hardness on the surface of the steel after heat treatment. An insufficient homogeneous diffusion time owing to the short duration of the rapid heat treatment process can be traced to non-uniformity in the properties of the quenched workpiece [21,22]. Damon et al. [23] investigated the effect of induction heat treatment on the uniformity of AISI M2 steel in the thickness direction; there were differences in hardness for different thicknesses, and the hardness of the steel stabilized at 230 HV at 6 mm from the surface layer after high frequency (HF) heat treatment. Zhang et al. [24] studied the change in the cross-sectional uniformity of steel plates and found that from 1/8 thickness direction to 1/2 thickness direction, the strength decreased from 659.4 MPa to 459.0 MPa. The analysis shows that fine-grain strengthening is the main reason for the change in strength. The effective grain size (EGS) at each thickness position is mainly related to ferrite/bainite in the thickness direction. EGS distribution across thickness positions exhibits a strong correlation with the ferrite/bainite phase distribution along the thickness direction, consequently influencing the through-thickness strength, toughness, and yield ratio of the steel plate.

The change in the cooling rate from the surface to the center results in the formation of polygonal ferrite and lath bainite with a non-uniform structure, both of which result in different surface and core properties of the steel plate [25]. Mechanical uniformity in bulb flat steel sections requires both uniform hardness distribution and consistent strength properties across the section but also the consistency of mechanical strength in critical regions, such as the bulb head and flat. Chen Xh et al. [26,27] adopted a VN micro-alloying technique to refine the structure for the non-uniformity in the cross-section of bulb flat steel, and reduced the difference in the strength of the bulb head and flat from 30~50 MPa to 5~10 MPa. However, the strength of bulb flat steel in the experiment is only 460 MPa class, and the discussion of the section hardness is missing. Regarding the 1.3 GPa bulb flat steel, Wang et al. [6] found bulb flat steel with a yield strength of 1292 MPa, an elongation of 18.5% and impact energy of 53 J at −196 °C by the QT process. However, only the flat strength variation was focused on; analysis of the bulb was missing, and even more attention was lacking about the section uniformity. Therefore, the study of the uniformity of the mechanical properties of the cross-section of bulb horizontal steel is of great importance.

The mechanical properties of high-strength bulb flat steel exhibit temperature sensitivity. This study systematically investigates the influence of the induction heating temperature on the microstructural evolution and mechanical properties of bulb flat steel, while elucidating the underlying mechanism for enhancing its sectional mechanical uniformity. Furthermore, a quantitative correlation between the prior austenite grain size and flat yield strength is established.

## 2. Experimental

### 2.1. Starting Material and Thermo-Mechanical Processing

**Figure 1:**
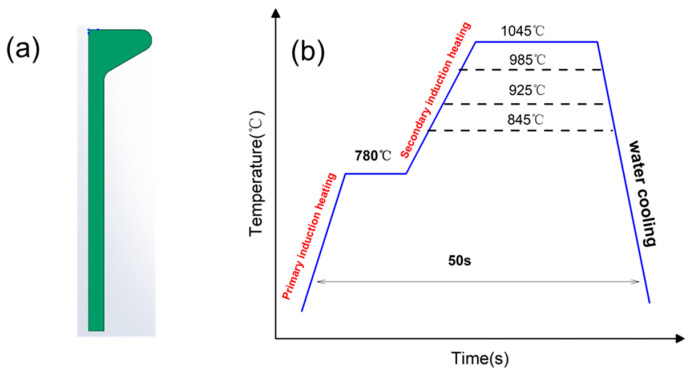
Hot-rolled bulb flat steel before quenching and quenching process diagram (**a**) Hot-rolled bulb flat steel (**b**) quenching process.

The test specimen was extracted from hot-rolled asymmetrical bulb flat steel (Grade No. 27) manufactured through an integrated production process including smelting, continuous casting, billet heating, and rolling. The cross-section of hot-rolled bulb flat steel is shown in Figure 1a. The chemical composition of the hot-rolled bulb flat steel was quantitatively analyzed using both spark discharge optical emission spectrometry (OES) and X-ray fluorescence spectrometry (XRF), with the detailed results presented in Table 1.

The thermal processing route for quenching is schematically presented in Figure 1b. Considering the substantial section modulus of bulb flat steel profiles, a sequential induction heating strategy is employed, comprising: (1) primary induction preheating to 780 °C for thermal equilibration, followed by (2) secondary induction heating to elevate the temperature to 845–1045 °C for complete austenitization.

During the heating process, adjusting the inductor power to achieve the set temperature is the most efficient method. The bulb flat steel is heated to 780 ± 8 °C by the first induction coil, and then the power of the secondary inductor is adjusted to 70% (T845), 80% (T925), 90% (T985), and 95% (T1045), respectively. After the secondary induction heating, each power corresponds to the stabilized surface temperature distribution of the bulb flat steel as shown in Table 2.

### 2.2. Microstructural Characterization

**Figure 2:**
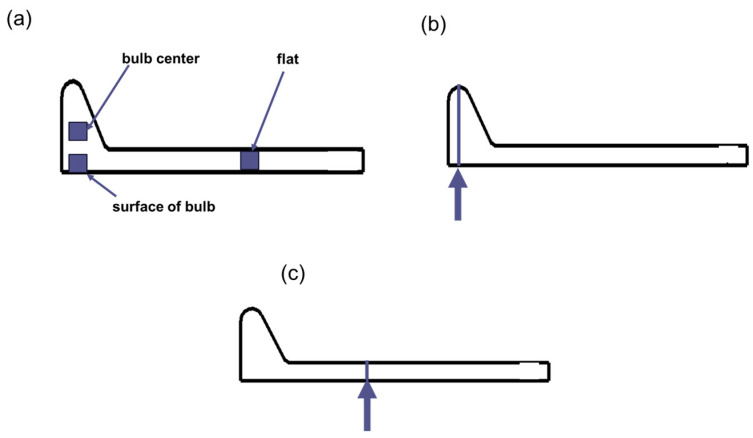
Bulb and flat hardness locations of bulb flat steel: (**a**) cross-sectional organization sampling location (**b**) bulb L direction (**c**) flat F direction.

The metallographic examination was conducted on a cross-sectional sample extracted along the thickness direction of the bulb flat steel. Following sequential mechanical grinding and polishing procedures, the specimen was etched using a 4% nitric acid—96% ethanol solution. Microstructural characterization was subsequently performed utilizing both optical microscopy (Olympus GX51 metallographic microscope) and field emission scanning electron microscopy (FE-SEM, JEOL JEM-7900F). Optical microscopic examinations were conducted on three distinct regions: the edge of the bulb, the core of the bulb, and the one-third position of the flat, as illustrated in Figure 2a.

Given that the bulb core exhibits the greatest thickness and, consequently, the slowest cooling rate, sampling was conducted specifically at this location. Throughout this study, unless explicitly stated otherwise, references to the “bulb” are indicative of the bulb core as the representative sampling area. For electron backscatter diffraction (EBSD) analysis, samples were extracted along the rolling directions of both the bulb and flat sections. These EBSD specimens were mechanically ground and subsequently prepared through electrolytic polishing to ensure optimal surface quality for microstructural characterization. The volume fraction of the electrolytic polishing solution was 10% perchloric acid and 90% alcohol solution, the electropolishing voltage was 18 V, and the polishing time was 20–25 s. The XRD samples were processed in the same way as the EBSD samples, and the dislocation density and austenite content measurements were carried out on an X-ray diffractometer (Bruker D8) using Co K-α radiation, 40 kV tube voltage, a scanning range of 45° to 115° scanning angle, a step size of 0.02°, and a scanning speed of 2°/min. The residual austenite content was calculated from the intensity of the XRD diffraction peaks [28]. The thin sheet, rolled along the bulb, was mechanically ground to 50 μm thickness using abrasive papers and subsequently punched into φ3 mm discs. Electrolytic thinning was performed at −25 to −20 °C using a solution of 7% perchloric acid in 93% ethanol. Microstructural characterization was conducted using field-emission transmission electron microscopy (FETEM, JEM-F200).

### 2.3. Mechanical Testing

The cross-sectional hardness of the bulb flat steel was measured using an HV-1000 Vickers hardness tester under a load of 5 kg and a dwell time of 20 s. The hardness measurements were conducted along two distinct orientations: the longitudinal direction of the ball head, denoted as the L direction in Figure 2b, and the transverse direction of the web, referred to as the F direction in Figure 2c.

**Figure 3:**
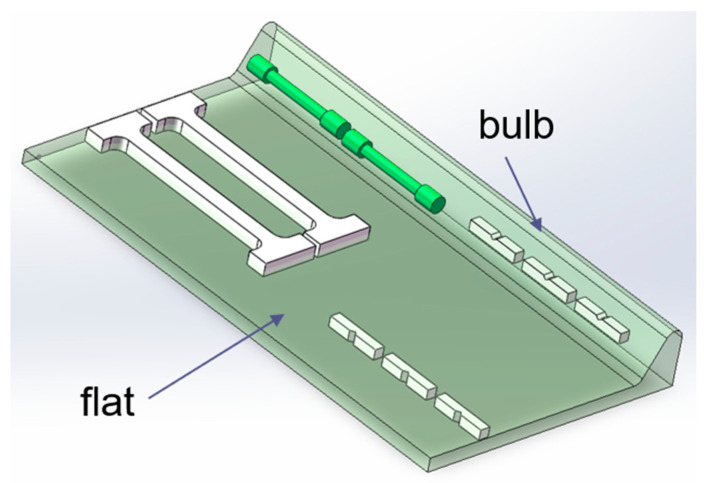
Tensile and impact sampling position and the sample shape.

Owing to the significant geometrical disparity between the bulb and flat sections, specimens were extracted from two distinct locations, the central region of the bulb and the one-third position of the flat. The specific sampling positions are illustrated in Figure 3. Room temperature tensile testing was conducted in compliance with the Chinese National Standard (GB/T 228) using a GNT200 universal testing machine [29]. The bulb section was machined into cylindrical tensile specimens with dimensions of M16 × 110 mm gauge length, while the flat section was processed into web tensile specimens to accommodate its geometrical constraints. The Charpy V-notch impact specimens were machined to the standardized dimensions of 10 mm × 10 mm × 55 mm in accordance with the Chinese National Standard (GB/T 229), featuring a 2-mm deep V-notch with a 45° angle and 0.25-mm root radius at the specimen center [30]. The preparation of the samples required for the various methods of study is shown in Table 3.

## 3. Results

### 3.1. Prior Austenite Grains

**Figure 4:**
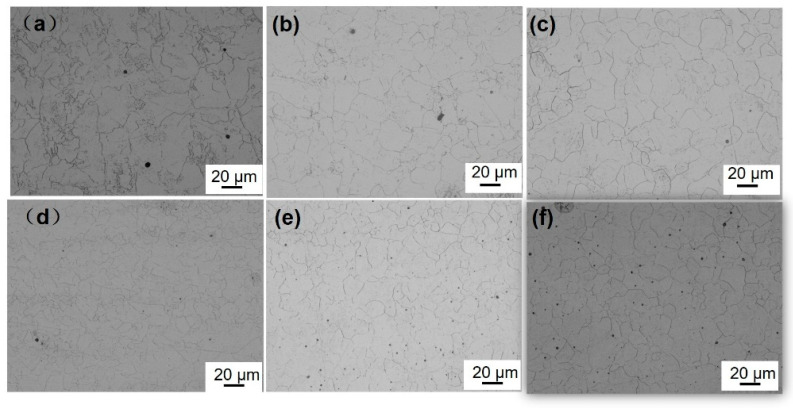
Optical micrographs of prior austenite grains: (**a**) bulb of T845 (**b**) bulb of T985 (**c**) bulb of T1045 (**d**) flat of T845 (**e**) flat of T985) (**f**) flat of T1045.

Figure 4 illustrates the prior austenitic grain morphology of the bulb flat steel following induction heating and quenching, with the heating temperature ranging from 845 °C to 1045 °C.

To examine the impact of the induction heating temperature on the initial austenite grain dimensions, 10 optical microscopy images were analyzed using the intercept method. The analysis revealed that the initial austenite grain size in both the bulb and the plate exhibited a progressive coarsening trend as the induction heating temperature was elevated. Induction heating to 845 °C resulted in insufficient homogenization of the austenite grain size. The prior austenitic grain size in the core region of the T925 bulb measured 13.72 μm. Upon increasing the quenching temperature to 1045 °C, a 15% grain growth was observed. In the case of T845 flat steel, the prior austenitic grain size was determined to be 9.69 μm. Notably, when subjected to the same elevated temperature of 1045 °C, the prior austenitic grain size in the flat section exhibited an increase from 9.91 μm to 13.03 μm. Comparative analysis revealed that within the same bulb flat steel specimen, the prior austenitic grain size in the flat region was significantly smaller than that observed in the bulb head section. These findings demonstrate the temperature-dependent grain growth behavior and microstructural heterogeneity in different regions of the steel components.

### 3.2. Microstructure

**Figure 5:**
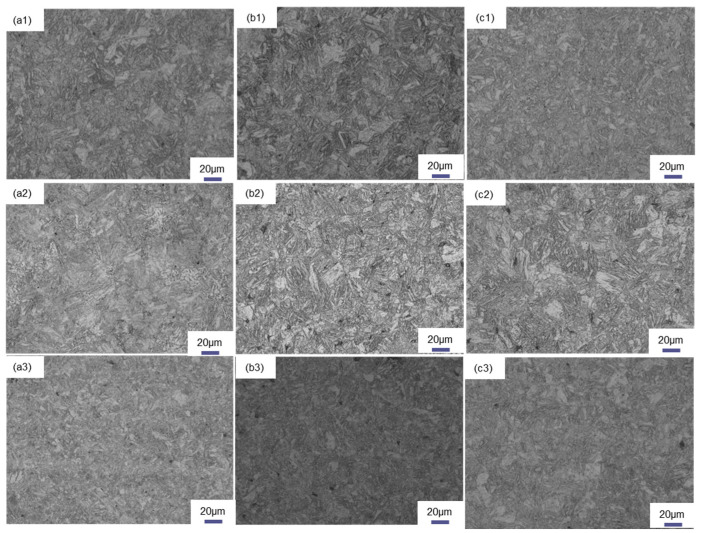
Optical micrographs of bulb flat steel sections at different induction heating temperatures: (**a**1–**a**3) T845 (**b**1–**b**3) T985 (**c**1–**c**3) T1045, (**a**1–**c**1) Surface position (**a**2–**c**2) bulb core position (**a**3–**c**3) flat position.

Figure 5 shows the optical micrographs of the cross-section locations of bulb flat steel with different induction heating temperatures, mainly containing martensite and bainite. The sampling location of the bulb flat steel cross-section is as shown in Figure 2a. Figure 5 shows that the quenched state bulbs are all mixed martensite/bainite in the heart and martensite in the flat.

**Figure 6:**
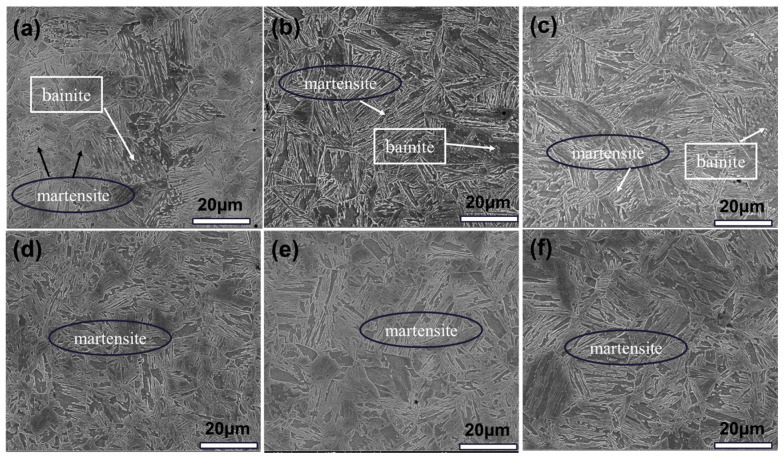
SEM morphology of bulb flat steel in quenched condition: (**a**) T845 bulb (**b**) T985 bulb (**c**) T1045 bulb (**d**) T845 flat (**e**) T985 flat (**f**) T1045 flat.

During the induction heating process and the subsequent rapid cooling stage, the limited diffusion kinetics of carbon atoms leads to a heterogeneous distribution of carbon within the austenitic matrix, thereby facilitating a phase transformation mechanism that promotes the simultaneous formation of ductile low-carbon bainite and high-strength martensitic structures [31]. The SEM morphology of the experimental steel with different induction heating temperatures is shown in Figure 6.

Regardless of variations in induction heating temperatures, the bulb cores consistently exhibited a dual-phase microstructure comprising both martensite and bainite, while the flat region demonstrated a completely martensitic microstructure. Upon elevating the induction heating temperature, the granular bainite microstructure in the core region of the bulb undergoes a progressive transformation into lath-shaped bainite. With a further temperature increase, the bainitic structure subsequently transforms into martensite through a directionless phase transformation process.

**Figure 7:**
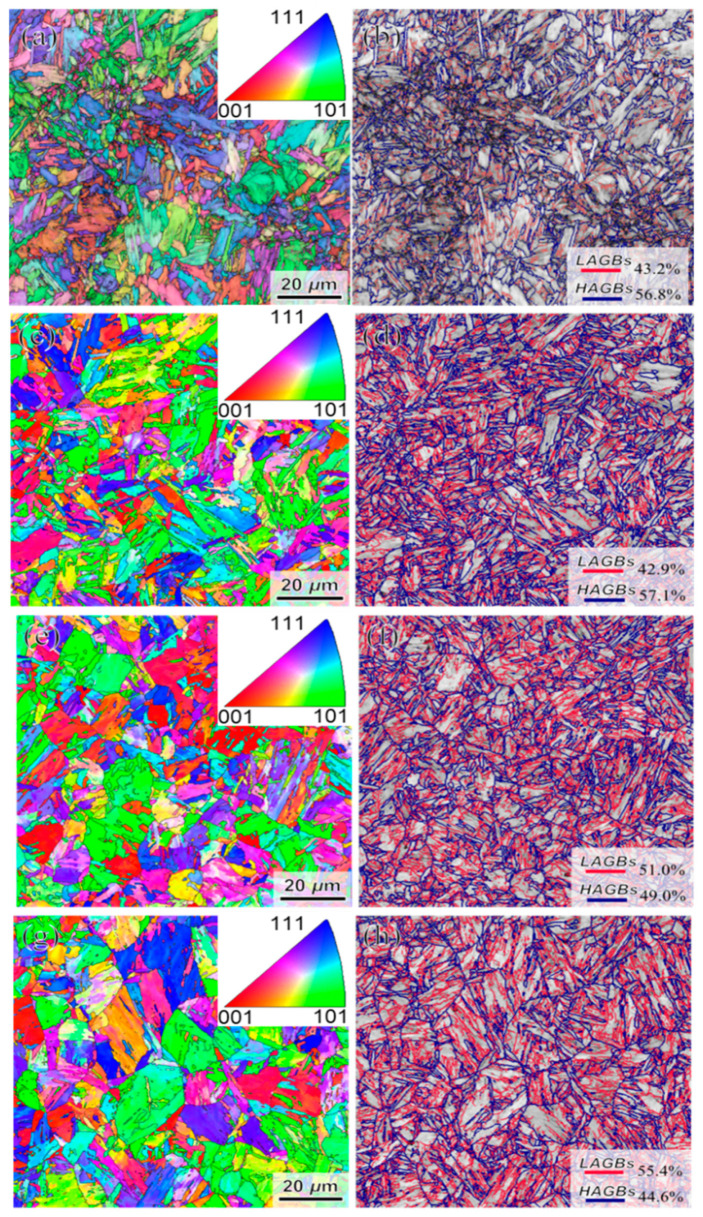
Inverse pole figure maps and phases of austenitized specimens at various temperatures, (**a**,**c**,**e**,**g**) T845/T925/ T985/ T1045 bulb IPF diagram (**b**,**d**,**f**,**h**) T845/T925/ T985/ T1045 for GB diagram.

The fine microstructure of the bulb core was characterized by EBSD, with the results presented in Figure 7.

The inverse pole figure (IPF) and grain boundary misorientation distribution at the central region of the bulb are presented in Figure 7. The red lines in the diagram indicate the distribution of low-angle grain boundaries (2°~15°), while the blue represents high-angle grain boundaries (>15°). Microstructural analysis reveals the presence of multiple blocks delineated by high-angle grain boundaries within the packet structure, while adjacent blocks maintain mutual contact through low-angle grain boundaries.

As the induction heating temperature increases, there is a tendency for the prior-austenitic grains in the heart to grow and for the morphological features of the laths to coarsen. The effective average grain size has been improved by 16% from 1.82 μm. Due to the relatively low induction heating temperature and the presence of carbides, the growth of austenite grains is effectively inhibited, resulting in the formation of a refined martensitic microstructure. However, as the induction heating temperature increases further, a significant coarsening of austenite grains occurs, leading to a reduction in high-angle grain boundaries, including prior austenite grain boundaries, packet boundaries, and block boundaries within the martensitic structure.

**Figure 8:**
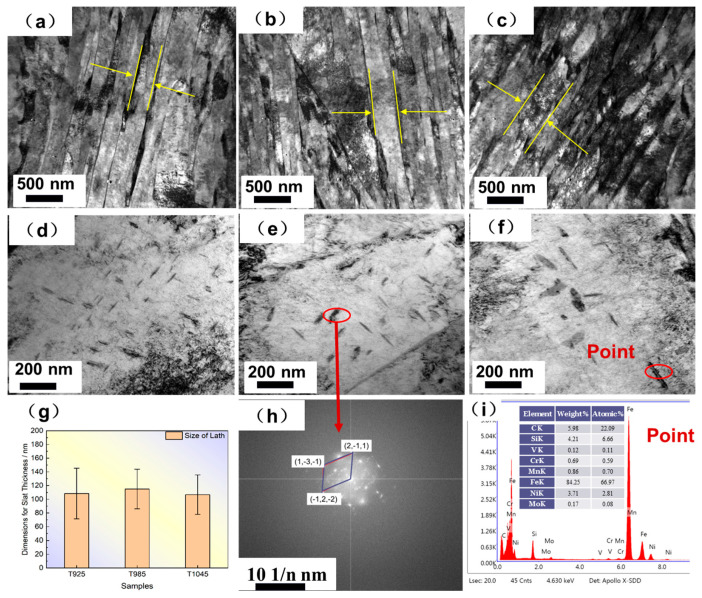
TEM images of hardened bulb: (**a**–**c**) T925~T1045 slat distribution (**d**–**f**) T925~T1045 precipitated phase distribution (**g**) Slat thickness distribution (**h**) Selected Area Electron Diffraction (SAED) pattern of the precipitated phase (**i**) Energy spectrum of the precipitated phase.

TEM is an important means of observing the fine structure of materials [2,8,32,33]. In order to observe the fine structure of bulb flat steel after quenching, transmission electron microscopy observation of the bulb heart was chosen. Figure 8a–c presents the bright-field TEM micrographs of various bulb core specimens, revealing distinct plate-like morphological features.

Furthermore, Figure 8d–f demonstrates the persistent presence of extensive dislocation networks within these plate-like structures, with carbide precipitates preferentially distributed along the plates. A total of 10 bright-field phase photographs were counted using the intercept method, and the average thickness of the slats was measured as in Figure 8g, with an average value of 110 nm for the T925 slat thickness, and an increase of 8 nm in the width of the slats when the temperature was increased to 1045 °C. Microstructural analysis reveals that the slat thickness exhibits temperature-dependent behavior, showing a progressive increase with elevated induction heating temperatures. Figure 8h,i shows the transmission morphology and EDS analysis of the T925, T985, and T1045 bulb cores, respectively, and it can be seen that the precipitated phases are predominantly of the M3C type by combining the EDS and selected electron diffraction. The morphology of the precipitated carbides exhibited a distinct elongation, with their average length demonstrating a significant increase from 79.8 ± 20 nm in the T925 condition to 111.2 ± 16.3 nm following the T1045 treatment.

### 3.3. Mechanical Property

**Figure 9:**
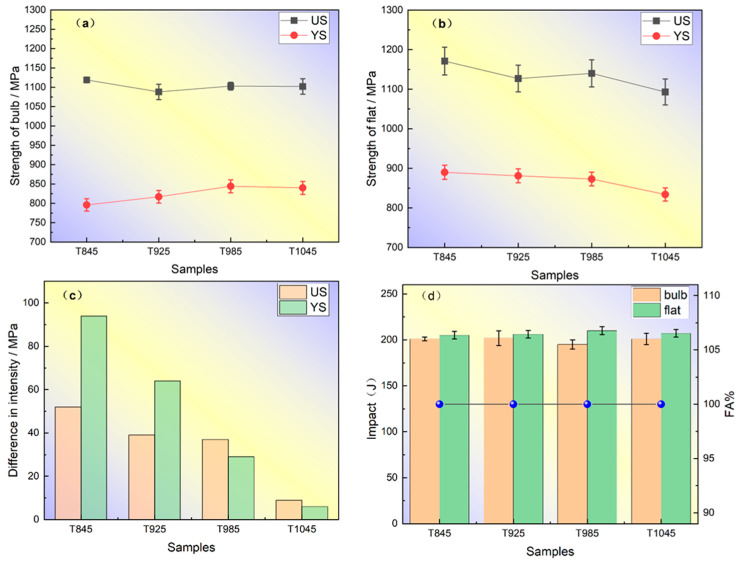
Effect of induction heating temperature on tensile and impact properties of bulb flat steel: (**a**) Tensile properties of the bulb (**b**) Tensile properties of the flat (**c**) Difference in strength between the bulb and the flat of the same bulb flat steel (**d**) Impact work of the bulb flat steel with varying process at −20 °C.

Figure 9 shows the relationship between the induction heating temperature and the effect of tensile and impact properties of bulb flat steel.

The experimental results indicate that the tensile strength (UTS) of the bulb head remained relatively stable within the induction heating temperature range of 845 °C to 1045 °C. However, the yield strength (YS) demonstrated temperature-dependent behavior, increasing from 796 MPa to 840 MPa as the temperature rose to 1045 °C, after which it reached a plateau and showed no significant further change with an additional temperature increase. The tensile and yield strengths of the flat plate exhibited significant temperature-dependent degradation behavior. When subjected to an induction heating temperature of 845 °C, the flat plate demonstrated a yield strength of 890 MPa. However, a substantial reduction in mechanical properties was observed with the increasing temperature, showing a 56 MPa decrease in yield strength when the temperature was elevated to 1045 °C. Similarly, the tensile strength of the flat reached 890 MPa at the elevated temperature of 1045 °C, indicating comparable temperature sensitivity in its mechanical performance. The strength of the flat is significantly higher than that of the corresponding bulb, and the strength difference between the flat and the bulb decreases with the increase in temperature. The strength difference between the bulb and flat is 94 MPa under the induction heating temperature of 845 °C, and the strength difference is reduced to 28 MPa when the induction heating temperature is further increased to 985 °C. The induction heating temperature was optimized to 1045 °C, resulting in a significant reduction in strength differential to 6 MPa. This represents a 93% decrease compared to the T845 condition, while simultaneously enhancing the cross-sectional strength uniformity of the spherical flat steel. Upon elevating the induction heating temperature from 845 °C to 1045 °C, the material exhibited consistent mechanical properties, with the elongation at break remaining constant at 16%. Simultaneously, the flatness retention was sustained at 14%, while the cross-sectional shrinkage of the bulb maintained a stable value of 73%.

**Figure 10:**
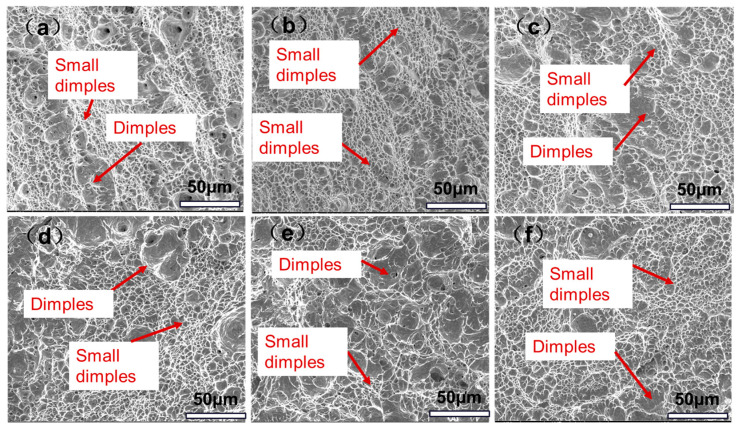
SEM photographs of impact fracture of bulb flat steel with induction heating temperatures from 845 °C to 1045 °C at −20 °C: (**a**) T845 bulb (**b**) T985 bulb (**c**) T1045 bulb (**d**) T845 flat (**e**) T985 flat (**f**) T1045 flat.

At −20 °C, the impact energy of both the bulb and flat sections for all four processes exceeded 190 J, with a fiber area percentage (FA) of 100% at the fracture surface. In addition, the difference between the impact function of the T1045 bulb and flat was only 6 J. Figure 10 displays SEM images of impact fractures in various bulb flat steels, showing a ductile dimple structure with large primary dimples surrounded by smaller secondary dimples. Micropores are visible in the radial zone, aligning with FA measurements and confirming the superior impact homogeneity of T1045 steel.

## 4. Discussion

### 4.1. Effect of Induction Heating Temperature on Prior-Austenitic Grains

**Figure 11:**
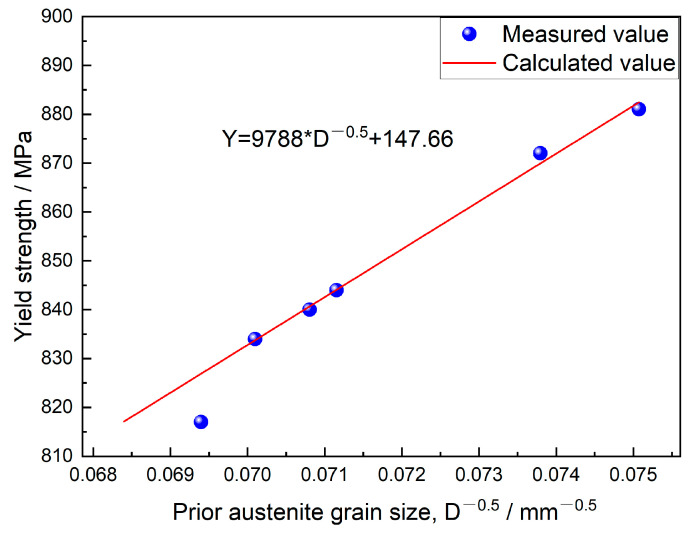
Relationship between prior austenite grain size and yield strength of the flat.

As the induction heating temperature rises, the prior austenite grain size grows due to enhanced atomic diffusion. After austenite forms, grain boundary atoms gain enough energy to migrate inward, causing grains to coalesce and grow [34]. Figure 4 shows that the flat section has significantly smaller prior austenite grains than the bulb section, highlighting the impact of geometry on grain growth. With bulb flat steel in the rolling process, the bulb size is larger, the temperature reduction is slower, the prior austenitic grain growth occurs, so the prior austenitic grain size of the bulb is larger than the flat. The prior austenitic grain morphology observed in the bulb region reveals that the T845 bulb exhibits irregular grain structure, attributed to insufficient austenitization caused by low induction heating temperatures. The alloy composition of the flat is maintained constant, with the quenching process yielding a complete martensitic transformation in the microstructure. The yield strength exhibits a significant dependence on grain size, following the classical Hall–Petch relationship which describes the inverse correlation between strength and grain diameter [35]. To quantitatively analyze this effect, the experimental data were systematically processed by establishing a mathematical relationship between yield strength and prior austenite grain size through coordinate system extrapolation. The quantitative relationship between the prior austenite grain size and yield strength is systematically presented in Figure 11.

The fitting results show that the overall fit of the regression line is R^2^ > 90%, and the calculated values are well fitted to the experimental. The mathematical relationship between the yield strength of the flat and the prior austenite grain size is:

(1) $$Y=1156.1*D^{-0.5}+519.7$$

*Y* is the yield strength, MPa; *D* is the original austenitic grain size, mm. It can be seen from the formula that the smaller the value of *D* is, the higher is the yield strength.

### 4.2. Effect of Induction Heating Temperature on Microstructural Evolution of Bulb Flat Steel

**Figure 12:**
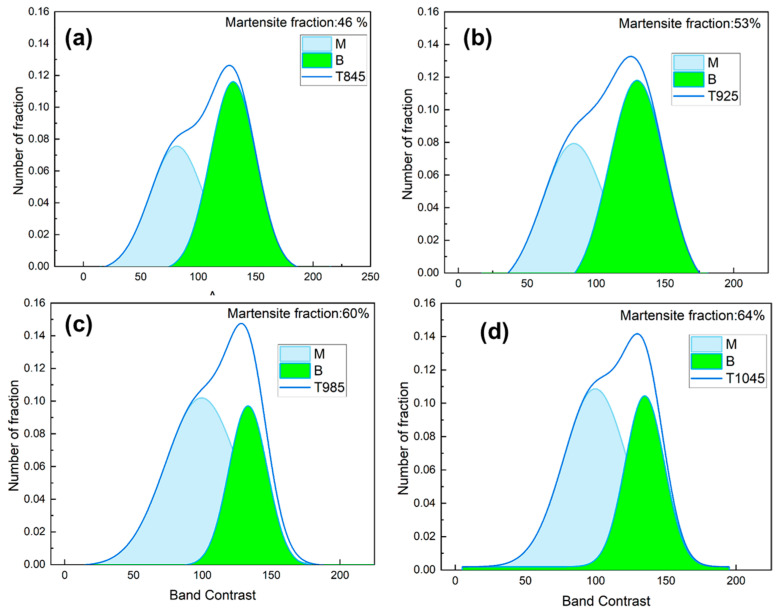
Effect of induction temperature on the organizational content of test steels: (**a**) T845 bulb martensite content (**b**) T925 bulb martensite content (**c**) T985 bulb martensite content (**d**) T1045 bulb martensite content.

Y. Wang et al. [36,37], reported that the difference in the strength of the Kikuchi pattern of martensite and bainite enables quantitative determination of martensite. Martensite has more defects such as carbon solutes, dislocations, and low angle boundaries compared to bainite, resulting in a relatively shallower Kikuchi pattern, leading to lower band contrast values, which are fitted to obtain the martensite content. Figure 12 illustrates the microstructural composition across various tissue regions within the bulb core. Quantitative analysis of martensitic transformation, as determined by BC values, reveals a significant temperature-dependent variation: As the induction heating temperature increases, the martensite content in T925 steel rises from 53% ± 5% to a stable level of approximately 60% ± 7%. A further temperature increase to the T1045 condition results in a martensite content of 64% ± 8%.

The hardness increases with the increase in the density of large-angle grain boundaries. Generally, martensite is harder than bainite, but its large-angle grain boundary density is lower than that of bainite [38].

The pearlite phase transformation is suppressed at higher temperatures, and the presence of alloying elements such as Ni and Mo reduces the generation of residual austenite, resulting in the presence of only martensite/bainite phases in the quenched state test steel. With the increase in the induction heating temperature, the proportion of martensite in the bulb region increases. During subsequent online quenching and cooling, the austenitic microstructure rapidly transforms into martensite. However, due to the relatively slower cooling rate in the core region, a portion of austenite transforms into bainite. Meanwhile, as the induction heating temperature increases, the driving force for austenite growth becomes more pronounced, leading to the migration of austenite grain boundaries and consequent coarsening of the original austenite grains. In these steels, hard phases (including martensite/bainite) and soft phases (including ferrite/austenite) often coexist. While increasing the content of hard phases can enhance strength, it typically results in reduced plasticity, representing the well-known strength–ductility trade-off [39,40].

### 4.3. Analysis of Cross-Sectional Hardness Uniformity in Bulb Flats

To facilitate the description of hardness variations across the cross-section of bulb flats, *T_S_* and *T_AV_* were employed as uniformity evaluation indices. A smaller *T_S_* value indicates reduced hardness fluctuations and improved hardness uniformity. The definitions of *T_S_* and *T_AV_* are given in Equations (2) and (3), respectively.

(2) $$T_{AV}=\frac{1}{m}\sqrt{\sum_{n=1}^{m}T_{n}}$$

(3) $$T_{s}=\frac{1}{m}\sqrt{\sum_{n=1}^{m}{{(T}_{n}-T_{AV})}^{2}}$$

In the formula, *m* represents the number of sampling points and *T_n_* denotes the hardness of the sampling points, HV5.

**Figure 13:**
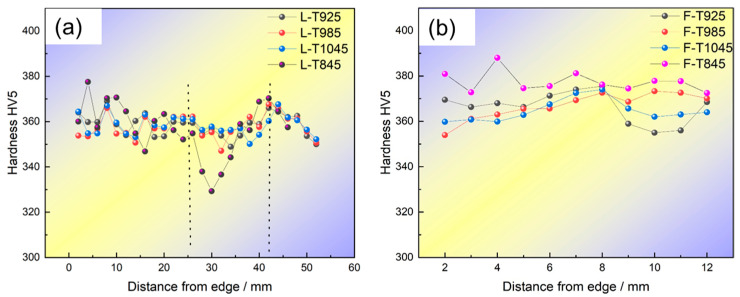
Effect of different induction heating temperature on hardness of quenched bulb flat steel section: (**a**) Bulb (**b**) Flat.

As illustrated in Figure 13a, the T845 specimen exhibits the minimum hardness value of 357 HV at the central region of the bulb, while demonstrating the poorest longitudinal uniformity with a variation range of 44.2 and a standard deviation of 11.9.

When the induction heating temperature exceeds 925 °C, a notable decrease in longitudinal hardness is observed with the increasing temperature. As the temperature is elevated to 1045 °C, the material exhibits a hardness of 356 *T_AV_* (Tensile Average Value) in the T1045 condition, with its tensile strength (*T_S_*) being approximately 50% of that measured in the T845 condition. This phenomenon suggests that an induction heating temperature of 1045 °C results in the most uniform longitudinal hardness distribution throughout the ball head component, as determined by microhardness measurements and tensile testing. Figure 13b shows the hardness distribution of the flat section. The flat hardness of T845/T925/T985/T1045 is 383/373/374/370, respectively. The flat hardness of T845 is the highest and the maximum range is 16 HV5. The microhardness analysis reveals that the T845 specimen exhibits the most significant hardness gradient between the bulb and flat regions, with a maximum differential of 30 HV5. This hardness distribution pattern correlates well with the observed strength variation trend. In contrast, the T1045 specimen demonstrates the smallest hardness differential of 14 HV5 between these regions, indicating a more homogeneous microstructure distribution. The T1045 flat bulb steel exhibits highly uniform microhardness distribution.

The thermal conduction gradient within the bulb results in a non-uniform hardness distribution, as evidenced in Figure 13a, where the hardness decreases progressively towards the center. This phenomenon is particularly pronounced in the T845 bulb, which exhibits the lowest core temperature, leading to the retention of the austenite phase and consequent reduction in hardness. Furthermore, the skin effect induces a deviation in the minimum hardness region from the geometric center of the ball head, creating an asymmetric hardness profile. The flat is harder than the bulb due to faster cooling and higher martensite content from its thinner section. Near the bulb’s rounded corner, the hardness decreases, likely because the sharp corner effect coarsens the austenite grains.

### 4.4. Effect of Induction Heating Temperature on Tensile Properties of Bulb Flat Steel Section

Previous studies have shown [36,41] that the yield strength of low-alloy steels can be attributed to a variety of strengthening mechanisms, including solution strengthening, fine-grain strengthening, dislocation strengthening, and precipitation strengthening [42,43]. The composition before induction hardening is the same as the heat treatment process, and the strength difference of the bulb flat caused by solution strengthening need not be calculated. In addition, if the induction hardening time is too short, the amount of precipitated phase is small, and the precipitation strengthening can be neglected. Therefore, it is only necessary to consider the effect of fine grain strengthening and dislocation strengthening on different positions of the bulb flat steel.

**Figure 14:**
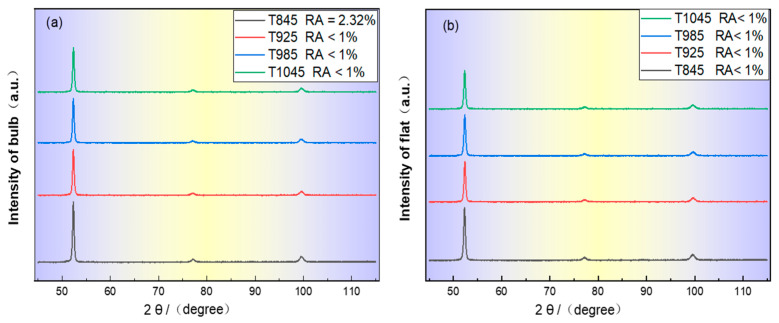
The effect of different induction heating temperatures on bulb flat steel XRD results: (**a**) Diffraction peak of bulb (**b**) Diffraction peak of flat.

Figure 14 is the XRD pattern of the bulb flat steel after quenching at different induction heating temperatures.

The calculated results show that the volume fraction of retained austenite in a T845 bulb is 2.32%, and that in other samples it is less than 1%. The limited austenite retention observed after rapid cooling can be attributed to two primary factors: On the one hand, the brief duration of induction heating and the relatively low concentration of austenite-stabilizing elements, particularly carbon (C) and manganese (Mn), within the cementite phase. However, at an induction heating temperature of 845 °C, a distinct microstructural evolution occurs. On the other hand, the core temperature of the bulb remains relatively low, leading to the preservation of residual austenite from the rolled state at specific locations, which persists through the subsequent quenching process.

The width of the diffraction peak was measured to obtain the dislocation density distribution [44] as shown in Table 4. It can be seen that the bulb/flat with an induction heating temperature of 1045 °C has the lowest dislocation density of 3.5 × 10^10^ cm^−2^/3.8 × 10^11^ cm^−2^.

The dislocation strengthening increment can be calculated using Taylor’s formula, such as Formula (4).

(4) $$\sigma_{d}=0.38Gb\sqrt{\rho }$$

For the contribution of dislocation strengthening, *G* is the shear modulus of the matrix, which is 76 (GPa), and *b* is the Berber vector, which is 0.248 (nm). *ρ* is the dislocation density of the matrix. With the increasing heating temperature, the dislocation strengthening contribution in the bulb initially rises and then declines, whereas in the flat region, it first decreases before slightly increasing. The corresponding data are presented in Table 5.

Based on the Hall–Petch calculation method, Equation (5) is used to calculate the contribution of fine grain strengthening:

(5) $$\sigma_{g}=K_{HP}\sqrt{d}$$

*K_HP_* is the Hall–Petch factor (0.21) and *d* is the effective grain size. Substituting the resulting effective grain size of 3.2 into Equation (5), the fine grain strengthening increment of the bulb flat steel is calculated as shown in Table 5.

It can be seen from Table 5 that the strength difference in the bulb and flat is mainly caused by dislocation strengthening when the induction heating temperature is 845~985 °C. When the temperature continues to rise, the strength difference is mainly close to the contribution of fine grain strengthening.

## 5. Conclusions

1. With the increase in the induction heating temperature from 845 °C to 1045 °C, a significant coarsening phenomenon is observed in the prior austenite grain size of both the bulb and flat sections. Quantitative analysis reveals that the prior austenite grain size in the bulb center at 1045 °C exhibits a 15.3% increase in coarseness compared to that at 925 °C. Furthermore, the prior austenite grain size in the flat section demonstrates a more pronounced coarsening effect, with a 31.4% increase relative to the grain size at 845 °C.
2. As the induction heating temperature is elevated from 845 °C to 1045 °C, the martensite volume fraction in the steel matrix demonstrates a significant increase from 46% to 64%, accompanied by the predominant precipitation of M3C-type carbides.
3. When the induction heating temperature ranges from 845 °C to 1045 °C, the strength of the quenched bulb exhibits an overall increasing trend, whereas the strength of the flat section demonstrates a gradual decrease with the rising temperature. Consequently, the yield strength differential between the bulb and flat sections is significantly reduced from 94 MPa to 6 MPa, resulting in a substantial enhancement in the mechanical uniformity across the bulb flat section.
4. When the induction heating temperature ranges from 845 °C to 985 °C, the disparity in yield strength between the bulb and flat components is predominantly attributed to dislocation strengthening mechanisms. However, at an elevated temperature of 1045 °C, the strength differential becomes more closely associated with fine grain strengthening effects.

## Figures and Tables

**Table 1:** Chemical composition of bulb flat steel (wt. %).

C	Si	Mn	P	S	Ni + Cr + Mo	V	Fe
0.10	0.23	0.54	0.006	0.0025	<6.0	0.06	Balance

**Table 2:** Temperature of spherical flat steel surface after adjusting secondary heating coil.

Secondary Coil Power Setting Ratio	70%	80%	90%	95%
temperature/°C	845 ± 5	925 ± 9	985 ± 6	1045 ± 13

**Table 3:** Methods of sample characterization and preparation.

Type of Sample	Method of Preparation
optical microscopy	Sanding, mechanical polishing, etching with 4% nitric acid and 96% alcohol solution.
SEM	Sanding, mechanical polishing, etching with 4% nitric acid and 96% alcohol solution.
EBSD	Sanding, mechanical polishing, etching with 10% perchloric acid and 90% alcohol solution.
XRD	Sanding, mechanical polishing, etching with 10% perchloric acid and 90% alcohol solution.
Tensile of bulb	Machined, dimensions M16 × 110 mm
Tensile of flat	Machined, dog-bone shaped, size 220 × 30 × 13 mm
Charpy V-notch impact	Machined, dimensions 10 × 10 × 55 mm

**Table 4:** Dislocation density of flat bulb steel quenched at different induction heating temperature.

Samples	Bulb (cm^−2^)	Flat (cm^−2^)
T845	3.2 × 10^11^	4.7 × 10^11^
T925	4.1 × 10^11^	5.9 × 10^11^
T985	4.2 × 10^11^	4.8 × 10^11^
T1045	3.5 × 10^10^	3.8 × 10^11^

**Table 5:** Contribution of strengthening mechanism to yield strength of bulb flat steel.

Samples	Position	σg (MPa)	σd	Yield Strength (MPa)
T845	bulb	143	404	796
	flat	147	489	890
Difference in strength(MPa)	-	−4	−85	−94
T925	bulb	155	457	817
	flat	172	548	881
Difference in strength(MPa)	-	−17	−91	−64
T985	bulb	121	465	844
	flat	168	498	872
Difference in strength(MPa)	-	−47	−26	−28
T1045	bulb	144	425	840
	flat	135	439	834
Difference in strength(MPa)	-	9	−12	6

## Data Availability

The original contributions presented in this study are included in the article. Further inquiries can be directed to the corresponding author.
